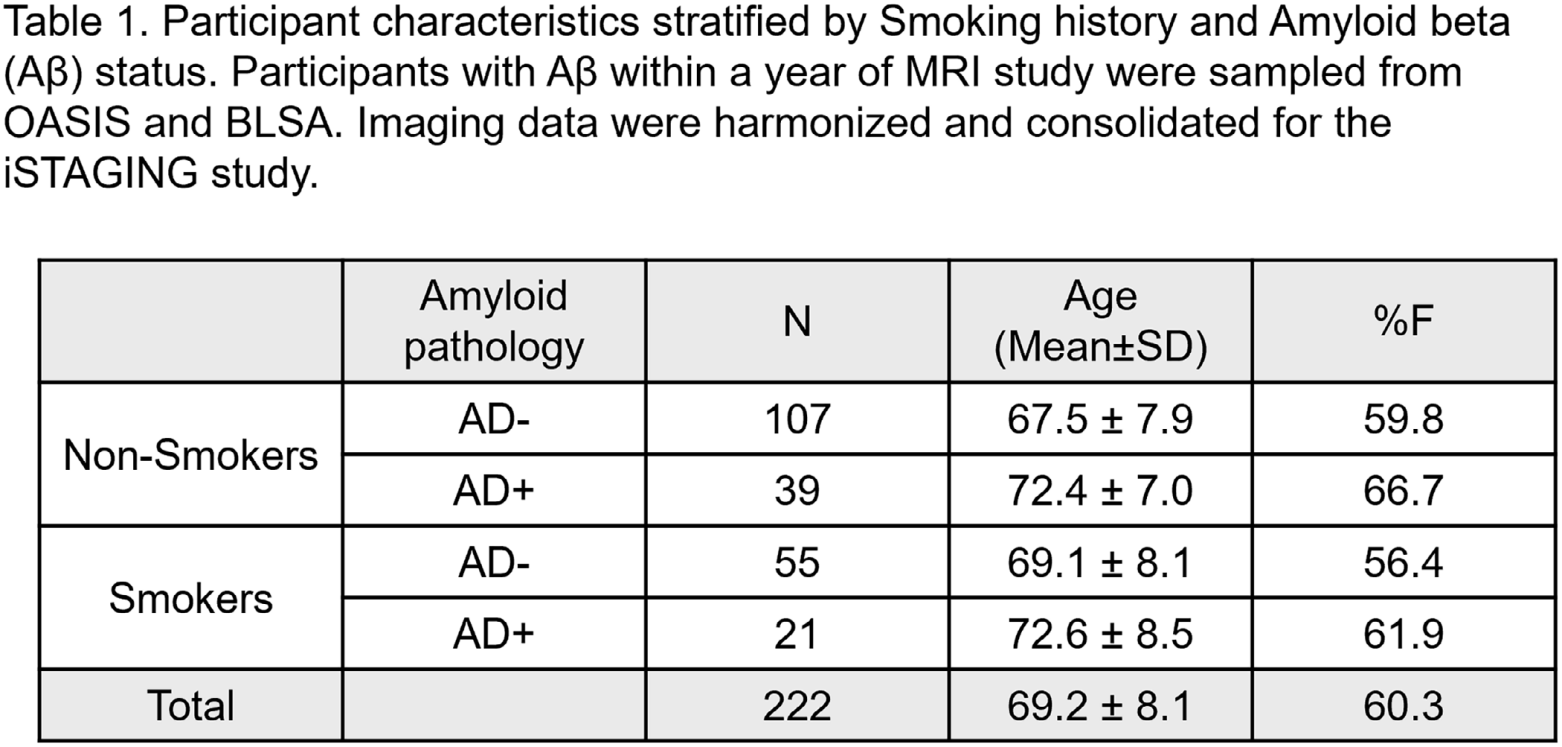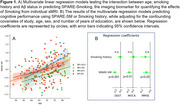# Investigating the combined effects of smoking and amyloid on brain structure in cognitively unimpaired adults using a machine learning‐based MRI marker

**DOI:** 10.1002/alz70856_107350

**Published:** 2026-01-09

**Authors:** Sindhuja Tirumalai Govindarajan, Elizabeth Mamourian, Dhivya Srinivasan, Guray Erus, Randa Melhem, Haochang Shou, Ilya M. Nasrallah, Christos Davatzikos

**Affiliations:** ^1^ Artificial Intelligence in Biomedical Imaging Laboratory (AIBIL), Center for and Data Science for Integrated Diagnostics (AI2D), Perelman School of Medicine, University of Pennsylvania, Philadelphia, PA, USA; ^2^ University of Pennsylvania, Philadelphia, PA, USA; ^3^ Artificial Intelligence in Biomedical Imaging Laboratory, Perelman School of Medicine, University of Pennsylvania, Philadelphia, PA, USA; ^4^ Department of Biostatistics, Epidemiology, & Informatics, University of Pennsylvania, Philadelphia, PA, USA; ^5^ Department of Radiology, University of Pennsylvania, Philadelphia, PA, USA

## Abstract

**Background:**

Smoking is a well‐established risk factor for cardiovascular disease, and its association with neurodegeneration and cognitive decline is an area of ongoing research. Critically, the interplay between smoking, Alzheimer's disease (AD) pathology, and cognitive impairment remains incompletely understood. This study investigated the relationship between smoking, AD pathology as indexed by amyloid‐beta (Aβ) deposition, and cognitive performance using SPARE‐SM, a novel machine learning‐based marker that quantifies smoking‐related spatial patterns of abnormalities on individual structural magnetic resonance images (sMRI).

**Methods:**

SPARE‐Smoking, derived from *N* = 37,098 cognitively unimpaired individuals from diverse cohorts, was evaluated in *N* = 222 individuals who had amyloid (Aβ) status available within +/‐ 1 year of the MRI scan in a subset of the training cohort. Amyloid deposition was determined using study‐specific cut‐offs for CSF and PET SUVR measures, categorizing participants as Aβ‐/Aβ+. Multivariable regression models were used to assess interactions between Aβ status, smoking history, and age on SPARE‐SM scores. Multivariable linear regression models, adjusted for age, sex, and years of education, examined associations between SPARE‐SM and cognitive performance.

**Results:**

While the proportion of smokers was similar between Aβ+ and Aβ‐ participants (Table 1), SPARE‐SM showed a nuanced relationship with both Aβ and smoking status (Figure 1A). Specifically, SPARE‐SM was higher than SM+Aβ‐ individuals in SM+ Aβ+ individuals (*p* <0.05) but lower in SM‐ Aβ+ individuals (*p* <0.05). Importantly, higher SPARE‐SM was associated with worse cognitive performance, whereas simply classifying individuals as smokers or non‐smokers showed no associations with cognitive outcomes (Figure 1B).

**Conclusion:**

These findings suggest a complex relationship between smoking, amyloid pathology, and cognition. The observation that SPARE‐SM differed by Aβ in smoking individuals highlights their potential synergistic effects on neurodegeneration. SPARE‐SM demonstrated associations with cognitive decline, even when clinical smoking status did not, emphasizing its potential for early risk identification. Further research is needed to disentangle the mechanisms linking smoking, brain changes, amyloid, and dementia.